# Causally-Rich Group Play: A Powerful Context for Building Preschoolers’ Vocabulary

**DOI:** 10.3389/fpsyg.2016.00997

**Published:** 2016-06-29

**Authors:** Jessie Raye Bauer, Amy E. Booth, Kathleen McGroarty-Torres

**Affiliations:** ^1^Department of Psychology, University of Texas at AustinAustin, TX USA; ^2^Northwestern UniversityChicago, IL USA

**Keywords:** word learning, causal information, causality, preschoolers, play-based learning

## Abstract

This work explores whether the facilitative effect of causal information on preschoolers’ word learning observed in the laboratory might be relevant to boosting children’s vocabulary in a group-play context. Forty-eight 3- to 4-year-old children learned six novel words for novel tools introduced during a small group-play session. Half of the groups used the tools according to their specified function to construct a fruit salad. The remaining children used the same tools to decorate a castle of blocks. In this way, some children learned about the causal properties of the tools, while others did not. Although children in both conditions comprehended the novel words equally well when tested shortly after the play session, learning in the Causal condition was more robust. Children’s comprehension scores in the Causal condition increased over time (a 7–20 day delay), such that children in this group performed better than children in the Non-Causal condition when tested in a follow-up session. These results demonstrate a striking benefit of causal enrichment to word learning in a context that could feasibly be implemented in preschool classrooms, playgroups, and individual households. Highlighting the causal properties of objects during playtime might offer a powerful approach to building children’s vocabulary, thereby providing a stronger foundation for early literacy and success in school more generally speaking.

## Introduction

Recent studies demonstrate a powerful facilitative effect of causal information (i.e., information that reveals the effective powers of an object or the nature of its contingent interactions with the environment) on learning in young children (Bauer and Fivush, 1992; Gopnik and Nazzi, 2003; Booth, 2008, 2009; Kemler Nelson et al., 2008; Cook et al., 2011). To date, however, all of the relevant evidence derives from observations of children interacting individually with an attentive adult in a highly controlled and distraction-free laboratory environment (e.g., Booth, 2009). It is therefore impossible to know whether the well-documented facilitative effect of causal information on learning has any relevance to real-world contexts. The current study begins to address this limitation by testing the influence of causal information on word learning in the context of small-group play.

We specifically focus on word learning in preschoolers because of its consequences for early literacy. Not only does early vocabulary strongly predict children’s vocabulary through much of grade school (Walker et al., 1994; Dickinson, 2001), it has serious implications for reading development (e.g., Cunningham and Stanovich, 1997; Muter et al., 2004; Dickinson et al., 2010). Moreover, preschooler’s vocabulary knowledge is marked by wide individual variability, with many children beginning school insufficiently prepared to take full advantage of their education (e.g., Hart and Risley, 1995). For these reasons, it is critical that we develop maximally effective strategies for facilitating the acquisition of vocabulary in young children.

Booth’s (2009) investigation of preschool word learning suggests one promising approach (see also Kemler Nelson et al., 2008). Three-year-olds in this study were taught novel words for six novel artifacts or animals. Some pictured items were additionally described in terms of their causal properties (e.g., functions) while others were described in terms of their non-causal properties (e.g., non-obvious perceptual features). Although children performed equivalently across the causal and non-causal conditions on a comprehension test administered immediately after training, children were more likely to remember the words taught in the causal condition after a delay of several days. One possible approach to efficiently building early vocabulary might therefore be to integrate causally rich semantic information about referents into word-learning activities.

Unfortunately, the relevance of the data supporting this recommendation to real world word-learning contexts is unclear. Participants in Booth (2009) learned words applied to individual pictures of imaginary novel objects in a one-on-one didactic context, free of competing distractions, that is highly uncharacteristic of children’s experience. More typically, children encounter new words applied to real three-dimensional objects in cluttered contexts and with other children (i.e., siblings, friends, or classmates) present (e.g., Fisher et al., 2014). In the current work we therefore evaluate whether causal information facilitates preschooler’s acquisition of novel words in a group play session. Small groups of children were taught novel names for six real tools while completing a play-based project under the direction of a highly trained member of our research staff. Half of the groups completed a project that allowed children to also learn about the causal properties of the novel tools (i.e., their functions) while the other groups completed an equally engaging project that did not reveal these causal properties. Participants also completed the Peabody Picture Vocabulary Test (PPVT), a normed receptive language test (Dunn and Dunn, 2007).

Because recent evidence suggests that causal information likely facilitates word learning by enhancing attention to, and therefore encoding of, the target material during training (Alvarez and Booth, 2014; Booth, 2015), the natural distractions that permeate a play-based learning environment might well diminish its influence. On the other hand, other potential mechanisms that affect the consolidation and retention of the target material, rather than the efficiency of its initial encoding, might be less disrupted by these circumstances. For example, Booth (2009) suggested that causal information might exert its influence in part by providing a framework for coherent elaboration of semantic information, thereby supporting the construction of memories for words and their meanings that are more robust over time. If children learn words more effectively in the causal than the non-causal condition, then this work will support the viability of causal enrichment as a strategy for increasing children’s vocabulary in real-world contexts. This work will therefore offer an important step toward translating basic research on the facilitative effects of causal information on learning to real-world educational practice.

## Materials and Methods

### Ethics Statement

This research was conducted according to all ethical guidelines provided by the American Psychological Association and with the approval of the institutional review board at Northwestern University. Parents/caregivers were presented with a detailed consent form before the study began. After parents signed the consent forms, children were escorted into the playroom. Children were allowed to be accompanied by parents/caregivers during the group session if they wished. Further, children were allowed to end participation at any time. The experiment involved a fun and engaging play session and activities for the children. In addition, the testing phases were conducted with a familiar research assistant and a stuffed animal.

### Participants

Forty-eight typically developing, native English-speaking preschoolers (*M*_age_ = 3.72 years, range = 3.1 to 4.4) were recruited through an established database of families interested in participating in developmental research. Twenty of these children were male (equally distributed across conditions). Training groups were created by unsystematically assigning children to an upcoming session that fit their schedule. Children were mostly White (85%), but some were Asian (9%), African American (4%), and Native Hawaiian (2%). None of the children were familiar with one another, with the exception of one set of twins (non-causal condition). There were no significant differences in PPVT-4 scores between the causal (*M* = 116.83, *SD* = 13.74) and the non-causal (*M* = 116.09, *SD* = 12.78) conditions. All but two mothers had earned at least a Bachelor’s degree. One mother had earned a High School Diploma (causal condition) and one had completed some college (non-causal condition). An additional nine participants (comparable in demographics to the full sample) were recruited, but ultimately excluded from analyses due to either failure to complete the experimental protocol (*n* = 4), hearing impairment (*n* = 1), or a strong response bias in which test pictures located in a single left, center, or right position were chosen on over 90% of test trials (*n* = 4). All participating children were given a book as a thank you gift, and parents were paid $20. This research was conducted according to all ethical guidelines provided by the American Psychological Association and with the approval of the institutional review board at Northwestern University.

### Materials

**FIGURE 1:**
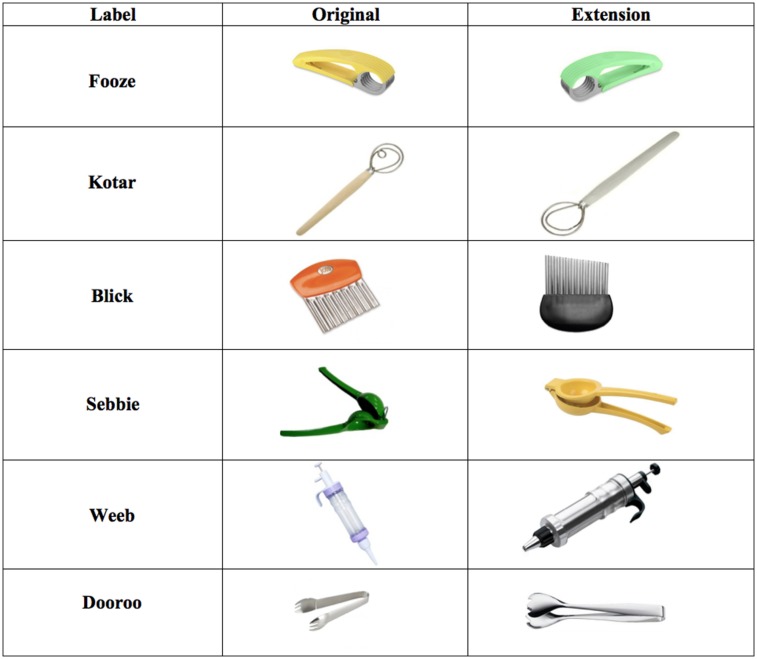
**Stimuli used during group-play activities and testing.** Objects presented during the group-play activity are shown in the first column. Pictures of these original training objects, as well as of the extension items, were presented during the test phase.

Six novel tools were selected based on their likely unfamiliarity to preschoolers and their ability to demonstrate a distinctive function in the context of making a yogurt-fruit salad (see **Figure 1**). These included a banana slicer, mini-tongs (used for picking cherries out of a small bottle), an icing pump (used to squirt yogurt onto the fruit), a lemon juicer, a dough whisk (used to scoop and strain peach halves out of a can of syrup), and a wavy chopper (used to cut peaches into textured slices). A photograph of each item was individually printed onto a 15.3 cm × 15.3 cm card. Each photograph also appeared in linear combination with two others on three different 55.8 cm × 15.3 cm cards for use in the testing phase. On these cards, images were spaced approximately 10 cm from each other. The order of photographs on these cards was determined in a constrained random manner, such that the correct target appeared in each position (left, center, right) an equal number of times and no picture appeared on more than two cards in a row.

An additional full set of these test cards was created picturing other exemplars from the same tool categories. These varied from the originals somewhat in color, size, and proportions (see ‘extension’ items in **Figure 1**). In total, 12 comprehension test cards were constructed.

### Procedure

Parents completed a brief demographic survey and rated their child’s familiarity with each tool (classified as: has used, knows the name of, has seen and/or has the object at home). Familiarity was categorized as either highly familiar (has used/knows the name of) or minimally familiar (has seen/has at home). After all parents completed and signed consent forms, children were escorted into a cheerfully decorated playroom in the laboratory. Although the number of children present and noise level in this setting clearly did not match that of a typical preschool classroom, the opportunities for distraction were much higher than in the stark experimental rooms used for individual training in prior work. In addition to the physical items within each child’s reach (bowls and spoons or blocks), other children were present in the room, and the walls were decorated with stickers. Much like in the iconic ‘circle time,’ the lead experimenter sat with her back to a blank wall in a circle with the children so that everyone had a clear view of her. Two other research assistants remained in the room to help facilitate the group activities. Children then completed the following procedure in which they were taught, and tested on their memory for, six novel words.

#### Training

Half of the groups (ranging in size from 4 to 7 children) were randomly assigned to a Causal condition (*M*_age_ = 3.74, *SE* = 0.07, *n* = 24, 15 female) in which children were introduced to the functions of six novel objects in the context of creating a yogurt-fruit salad. Although there are a number of ways in which we might have instantiated causal information, we chose to focus on object functions here because they embody particularly rich causal relations between goals, actions, object properties, and outcomes in the domain of artifacts (Bloom, 1996; Booth, 2006). Children stood around a low bean-shaped table to complete this activity. After a brief introduction to the task, the experimenter named and demonstrated the function of each tool individually. For example, she said, “This is a kotar. Kotars are used to scoop peaches from syrup! Watch me use this kotar.” Children then each got a turn to try out the tool themselves to assemble their own snack. This procedure therefore not only exposed children to the novel target words, but also gave them an opportunity to observe and try out the functions of the tools labeled by those words.

The remaining groups were assigned to a Non-Causal condition (*M*_age_ = 3.71, *SE* = 0.06, *n* = 24, 13 female) in which children decorated a castle of various types of blocks under the guidance of the experimenter. Children sat on the floor around a large Duplo-block building surface. After a brief introduction to the task matched in length and structure to that used in the Causal condition, the experimenter named and described each target object individually while demonstrating its full range of motion. For example, she said, “This is a kotar. Kotars have a long handle and are swirly at the end! Think about where we can put this kotar on the castle.” Children then each got a turn to manipulate the tool and place it as a decoration on the castle before the experimenter decided on the best final spot to leave it.

Importantly, while this group-play task was, on the surface, quite different from the Causal project, it was comparable in several important ways. First, the activities central to both conditions were highly engaging to children. Indeed, coders blind to the experimental hypotheses were unable to detect any differences in engagement (rated on a five point scale) across groups (*M*_causal_ = 4.41, *M*_non-causal_ = 4.57). Second, the two conditions both specifically highlighted the shape of the objects. In the Causal condition, this was achieved implicitly through the demonstration of object functions while in the Non-Causal condition it was achieved through explicit description followed by placement on the castle in locations that conformed to those shapes. Third, both conditions offered equal opportunities for children to physically interact with and manipulate the objects. Each child was given a turn to use the tool in a goal-directed manner either to make their fruit salad (Causal condition), or to place the object in a special place on the castle (Non-Causal Condition). Fourth, the two conditions provided equal exposure to the novel target words. All groups (regardless of size) heard the novel words the same number of times. Children rarely repeated or otherwise spontaneously produced the novel words, and these instances occurred with equal frequency in the Causal and Non-Causal conditions. Finally, the two groups were matched on age (*M*_causal_ = 3.74, *M*_non-causal_ = 3.71) and group size (*M*_causal_ = 5.58, *M*_non-causal_ = 5.75), and the sex composition of the Causal (*n* = 15 females) and Non-Causal (*n* = 13 females) groups was comparable. Thus, the key difference between the Causal and Non-Causal conditions was in children’s opportunity to learn about the causal properties (i.e., functions) of the novel tools.

Children from each condition concluded the group-play session with a snack. Those in the Causal condition ate their yogurt-salad while those in the Non-Causal condition ate a store-bought snack of their choice (e.g., goldfish crackers). While the children ate, the experimenter reminded the children of all the new things they just played with by holding up each novel object individually and repeating its name and description. For example, in the Causal condition, she said, “Today we saw this kotar. Kotars are used to scoop peaches out of syrup. Remember, this is called a kotar.” In the Non-Causal condition, she similarly reiterated the name and distinctive physical property of each object.

#### Testing

Children were escorted individually into another quiet room for testing where they were introduced to a stuffed animal that wants to learn all about what they just did in their group-play session. Children first taught the animal about the words they learned by playing the ‘pointing game’. In this forced-choice selection task, children demonstrated their basic understanding of each novel word by pointing to its referent from among pictures of three items presented during training. In order to familiarize children with this task, the experimenter first presented a card picturing three familiar items (e.g., an apple, a shoe, and a cat) and asked the child to point to the shoe. Test cards picturing the novel stimuli were then presented in a fixed order across subjects, and labels were tested in the same order as they were introduced during the group-play session. Test cards were presented such that the ‘correct’ picture appeared in the left, right, and center position an equal number of times. Also, any single picture never appeared for more than two consecutive trials, and when it appeared twice in a row, its position was changed. For each trained label, children were asked to find its referent on the corresponding test card (e.g., “Can you point to the _______ ?”). Because comprehension requires not only mapping a new word to its trained referent, but appropriate extension of that word to novel referents, we then repeated this task using test cards that pictured new examples of the potential referents (see extension items in **Figure 1**). Thus, children experienced a total of 12 comprehension test trials (one trained mapping and one extension trial) for each novel word they heard during the play session.

#### Follow-up Testing

Children returned one to 3 weeks (*M*_causal_ = 10.67 days *SD* = 3.08, *M*_non-causal_ = 10.13 days, *SD* = 3.11) later to repeat comprehension. The same procedure was used except that a different curious stuffed animal was introduced as pretense for playing the games. The PPVT-4 was then administered according to standard procedures (Dunn and Dunn, 2007).

### Coding

A primary coder, who was blind to condition, recorded the choices made during comprehension testing by each subject on each trial. They did so by viewing the test phase only of the recorded experimental sessions and assigning a point for each correct selection. A secondary coder, who was also blind to condition, independently coded the responses of 25% of the subjects. Coders agreed on 98% of comprehension trials.

## Results

Preliminary analyses revealed no main effects of, or interactions with, gender [*F*(1,42) = 3.16; *p* = 0.08], test type (mapping to original target or extension item) [*F*(1,95) = 1.11; *p* = 0.29], group size [*F*(1,42) = 3.52; *p* = 0.07], number of days between testing sessions [*F*(1,42) = 2.28; *p* = 0.14], or age [*F*(1,42) = 0.05; *p* = 0.82] on word-learning performance, so we collapsed across these variables in all of the analyses reported here. Finally, we did not include object familiarity in our analyses as parents rated an average of less than one object (out of 6) as highly familiar to their child. There were also no significant differences in level of familiarity between conditions [highly familiar: *M*_causal_ = 0.38, *M*_non-causal_ = 0.29, *t*(46) = 0.48; *p* = 0.63; minimally familiar: *M*_causal_ = 1.21, *M*_non-causal_ = 0.75, *t*(46) = 1.46; *p* = 0.15], suggesting familiarity did not significantly influence performance.

**FIGURE 2:**
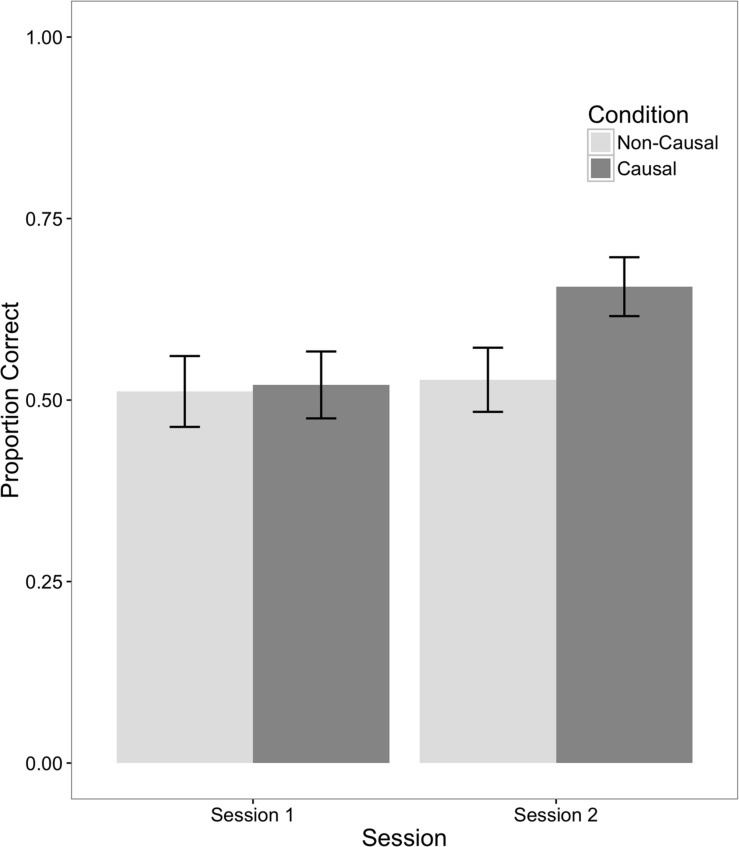
**Means and standard errors of the proportion of word comprehension trials on which the correct referent was chosen in each condition during each testing session**.

We began by tabulating, for each child, the proportion of test trials on which they identified the correct referent during the first and second testing sessions. One sample *t*-tests showed average performance was above chance in both conditions during both testing sessions (*p*s < 0.01), suggesting that children understood and were engaged in the task. We therefore proceeded to evaluate the potential effect of training condition (Causal vs. Non-Causal) on comprehension. Although we initially considered Comprehension Test Type (trained mapping vs. extension) as a within subject variable, no main effects or interactions were evident, so performance across both types of comprehension trials was collapsed in the final analyses. A mixed model ANOVA including Condition (Causal vs. Non-Causal) as a between subject factor and Session (first vs. second) as a within subject factor (see **Figure 2**) revealed a significant main effect of Session, *F*(1,46) = 6.79; *p* = 0.01, *d* = 0.34, reflecting overall better word-learning performance in the second than in the first session. This main effect of Session was further qualified by a significant interaction with Condition, *F*(1,46) = 4.21; *p* = 0.046. We therefore proceeded to compare the two experimental conditions at each measurement time point. No difference in performance was observed during Session 1 (*M* = 0.52, *SE* = 0.05 vs. *M* = 0.51, *SE* = 0.05). However, children were able to identify the referents of more words in the Causal (*M* = 0.66, *SE* = 0.04) than the Non-Causal (*M* = 0.53, *SE* = 0.04) condition when tested again in Session 2, *t*(46) = 2.14; *p* = 0.04, *d* = 0.62. This difference was mirrored in a non-parametric analysis. More children in the Causal condition (*n* = 17) than in the Non-Causal condition (*n* = 9) responded correctly on more than half of the test trials in Session 2, Fisher’s exact *p* < 0.05.

## Discussion

Our goal in this investigation was to test the generalizability of laboratory-based evidence of causal information’s facilitative effect on early word learning to group-play contexts. Despite the substantial increase in uncontrolled distractions that this environment introduced, children were more successful at learning words when the causal properties of labeled referents (i.e., object functions) were made evident as part of the group project than when the same objects were incorporated in a non-causal way.

It is important to note that word-learning performance was actually initially equivalent across conditions, with children in the Causal condition outperforming those in the Non-Causal condition only after a lengthy delay. This pattern of deferred influence mirrors precisely that observed in Booth (2009). Booth offered two potential explanations for this distinctive effect, favoring the possibility that the influence of causal information on performance at the time of initial testing was simply masked by the fact that children were fatigued at that point, having just completed a lengthy training procedure. This explanation fit well with the fact that performance in both conditions failed to rise above chance at the time of initial testing in that work.

However, in the current study, children performed significantly above chance in both conditions at the time of initial testing. This was perhaps due to the relatively fun atmosphere, the opportunity to interact with three-dimensional objects (as opposed to pictures), and the insertion of a substantial break (including a drink and snack) between training and testing. Regardless, the strong level of initial performance observed here, rules out fatigue as a viable explanation for the current findings. Booth alternatively suggested that the delayed effect of causal information on learning might derive from its influence on the consolidation of semantic memories over time. Although specifying the precise mechanisms underlying this mode of influence will require further investigation, existing research already suggests some possibilities. For example, evidence suggests that consolidation during sleep is integral to vocabulary learning in children (Henderson et al., 2012), and that retention of abstract relational information, perhaps like that embodied in causal properties, reaps particular benefit from sleep and even brief naps (e.g., Stickgold and Walker, 2004; Wagner et al., 2004; Gomez et al., 2006).

Importantly, evidence for an influence of causal information on long-term consolidation does not negate the possibility that it also influences initial encoding of words and their meanings. For example, given research and theory suggesting the dominance of shape in helping young children identify the boundaries of novel categories (e.g., Landau et al., 1988; Graham et al., 1999; Ware and Booth, 2010), one possibility is that demonstrating causal powers of objects implicitly highlights the shapes that constrain those demonstrations (e.g., the circular opening on a fooze into which bananas must be inserted to be sliced). In the current design, however, we undermined this potential advantage by explicitly describing the global shape and/or part structure of target objects in the Non-Causal condition.

Recent evidence also suggests that children are particularly motivated to learn about the causal properties of animals and artifacts (Kemler Nelson et al., 2004; Greif et al., 2006; Alvarez and Booth, 2014), and thus might pay more attention to this type of information in learning tasks. Indeed, children require fewer training trials to encode causal descriptions of novel items, as well as labels for those items, than they require for non-causal descriptions and associated labels (Booth, 2015). Thus, demonstrating the causal powers of labeled objects might facilitate learning by heightening children’s attention during training. Attention levels and apparent interest in the group-play tasks was high across participating children. Indeed, almost every child was highly attentive and fully participatory throughout the play period, and those who were not, were similarly distributed across conditions. One child in each condition was too shy to join the activity and merely observed with their parent from the side. Another two children in the Causal condition, and one in the Non-Causal condition, observed the beginning of the session, but then actively joined the group. It may be that the influence of causal information on attention and initial encoding will only be evident when tasks are less compelling in and of themselves.

Further, children manipulated the objects with high levels of enthusiasm in both conditions, as reflected in our coding attentiveness and engagement. While the mechanics of children’s object manipulation did differ across conditions, they were well matched in several key respects. Children manipulated the objects for equal amounts of time across conditions, and did so in a way that was both goal directed (building the fruit salad or decorating the castle) and constrained by the physical properties of the object. That is, each object was well suited for prepping one particular type of food for the fruit salad and for balancing on particular parts of the castle. What differed is the degree to which the manipulation revealed the causal powers of the object. Although differences in attention could not be detected in our coding of observable behavior, both of these mechanisms could have been active at a cognitive level.

Whatever the mechanism(s) prove to be, the current work adds to a now substantial body of evidence demonstrating the powerful role that causal information plays in facilitating learning in preschoolers. Importantly, it adds to this literature by demonstrating that this influence extends to at least one group-learning context. Small-group play that is rich in causal information can be practically implemented by teachers in preschool center activities, caregivers in daycare settings, as well as by parents in children’s homes. That said, the current work represents only an initial step toward fully translating our laboratory-based knowledge of causal supports for learning into educational practice.

Would the influence of causal information remain strong in a noisy Head Start preschool classroom? Could it be usefully integrated into whole-class ‘project play’ activities or story time? Will the effect generalize to production, or to more rigorous tests of comprehension, if training is more fully integrated into a child’s daily activities? Is this phenomenon relevant to learning real words in a classroom where children might have varying levels of familiarity with the target words? It is important that future research tests the generalizability of these results to more socioeconomically and culturally diverse populations, as well as to other learning contexts that vary in the degrees of playful versus didactic interaction, and amount and types of distractions.

Our hope is that the current work will inspire further investigation into the full range of contexts and conditions under which causal information facilitates children’s learning. In so doing, we can collectively articulate optimal ways to harness the power of causal information for promoting early learning of vocabulary, a goal fundamental to school readiness and longer-term literacy outcomes.

## Author Contributions

AB designed the study. KM-T collected and JRB and AB analyzed the data. JRB and AB collaboratively wrote the manuscript and JRB provided additional critical revisions. Both JRB and AB have approved the final version of the manuscript and agree to be accountable for all aspects of the work.

## Conflict of Interest Statement

The authors declare that the research was conducted in the absence of any commercial or financial relationships that could be construed as a potential conflict of interest.
